# The changing seroepidemiology of enterovirus 71 infection among children and adolescents in Singapore

**DOI:** 10.1186/1471-2334-11-270

**Published:** 2011-10-11

**Authors:** Li W Ang, Meng C Phoon, Yan Wu, Jeffery Cutter, Lyn James, Vincent T Chow

**Affiliations:** 1 Epidemiology and Disease Control Division, Ministry of Health, College Road,169854, Singapore; 2 Department of Microbiology, Yong Loo Lin School of Medicine, National University of Singapore, Kent Ridge, 117597, Singapore; 3 Communicable Diseases Division, Ministry of Health, College Road, 169854, Singapore; 4 Office of Deputy Director of Medical Services (Public Health), Ministry of Health, College Road, 169854, Singapore

## Abstract

**Background:**

Enterovirus 71 (EV71) has caused recurrent epidemics of hand, foot and mouth disease among children in Singapore. Between August 2008 and July 2010, we conducted a survey to estimate the seroprevalence of EV71 infection among children and adolescents aged 1-17 years. We compared our EV71 seroepidemiologic findings with a previous study conducted in 1996-1997.

**Methods:**

The survey involved the prospective collection of 1,200 residual sera from Singapore residents aged 1-17 years in two hospitals. Neutralizing antibodies to EV71 were detected by the microneutralization test. The geometric mean titer (GMT) of EV71 antibodies and 95% confidence intervals (CI) were calculated and compared by age groups. Statistical significance was taken as *P *< 0.05.

**Results:**

The overall EV71 antibody prevalence was 26.9% (95% CI: 24.5-29.5%). It increased significantly from 14.3% in children aged 1-6 years to 27.8% in those aged 7-12 years, and reached 38.8% in adolescents aged 13-17 years. The seroconversion rate differed by about 12% between the consecutive age groups. The GMT of EV71 antibodies was higher among primary school children aged 7-12 years in our study than that among the 6-12 year age group in the 1996-1997 study.

**Conclusions:**

Higher antibody titers were observed in children aged 1-6 years than those in the other two age groups, indicating that most of the infections had been acquired during early childhood. EV71 infection is common among children and adolescents in Singapore, with 39% infected by the time they are in secondary school (13-17 years of age).

## Background

Enterovirus 71 (EV71) and coxsackievirus A16 (CA16) have caused large epidemics of hand, foot and mouth disease (HFMD) worldwide. Since EV71 was first identified in 1969 from an infant suffering from encephalitis in California [1], outbreaks associated with this virus have been documented, including in Australia in 1972, Japan in 1973 and 1978 [2,3], Bulgaria in 1975 [4], and Hungary in 1978 [5]. EV71 infection is occasionally associated with severe complications (such as encephalitis) and deaths in children. Since 1997, EV71-related HFMD epidemics in the Asia-Pacific region have been increasingly reported, including in Sarawak, Malaysia in 1997 [6], 2000 [7,8], 2003 and 2006 [9]; Brunei in 2006 [10]; Perth, Australia in 1999 [11]; Taiwan in 1998 [12] and 2000 [13]; Japan in 2000 and 2003 [14]; and China in 2008 [15].

In Singapore, HFMD was first recognized in an outbreak in June-July 1970, but the etiologic agent was hitherto unknown. CA16 was associated with two other outbreaks without serious complications or fatalities, involving 104 individuals between September 1972 and January 1973, and 742 individuals between September and December 1981 [16,17]. EV71 was first isolated from an infant with symptoms of HFMD in Singapore in 1984. Between September and October 2000, a large EV71-associated HFMD outbreak occurred in Singapore, resulting in 4 deaths [18,19]. HFMD became notifiable under the Infectious Diseases Act from 1 October 2000. All preschool centers were closed from 1 October to 15 October 2000. By 28 October 2000, a total of 2,827 cases were notified. The main pathologic findings in the fatal cases were encephalitis, interstitial pneumonitis, and myocarditis. Thereafter, EV71-associated HFMD epidemics occurred in 2006 and 2008, with the latter being the largest known HFMD outbreak in Singapore [20].

HFMD is endemic in Singapore, and more than 50% of cases occur in children below 5 years of age. Although the predominant circulating enteroviruses change periodically, the two major enteroviruses causing nationwide HFMD epidemics in Singapore have been CA16 and EV71 [21]. An EV71 serologic survey in Singapore had been conducted on serum samples collected from 856 children aged 12 years or younger at a pediatric clinic at the National University Hospital (NUH) between July 1996 and December 1997 [22]. All children who were born at the hospital or brought for routine visits and vaccinations during this 18-month period were included, and they did not exhibit HFMD-related symptoms at the time of sample collection. Since then, there had not been any comprehensive survey to measure the EV71 seroprevalence between or after EV71-associated HFMD epidemics in Singapore.

Between August 2008 and July 2010, we conducted a seroprevalence survey to estimate the levels of EV71-specific neutralizing antibodies among children and adolescents aged between 1 and 17 years. This was the largest and second nationally representative survey conducted to ascertain the latest age-specific seroprevalence of EV71 infection in Singapore. We compared our findings with the results of the 1996-1997 study to discern any significant changes over the past decade.

## Methods

### EV71 seroprevalence study design

The Ministry of Health (MOH) conducted a national pediatric seroprevalence survey between August 2008 and July 2010 involving the prospective collection of residual sera following the completion of routine biochemical investigations by diagnostic laboratories in KK Women's and Children's Hospital and NUH. This survey was carried out in accordance with Section 7 of the Infectious Diseases Act which provides for the use of residual samples for the purpose of public health surveillance.

Sera of Singapore citizens and permanent residents who were ethnic Chinese, Malay and Indian aged between 1-17 years attending inpatient services or day surgery were collected. Patients were excluded if they were known to be immunocompromised, on immunosupressive therapy, or had been diagnosed with measles, mumps, rubella, chickenpox, diphtheria, pertussis, poliomyelitis, hepatitis B, dengue or HFMD.

On the premise of an anticipated EV71 seroprevalence of 33% in each of the age groups of 1-6 years, 7-12 years and 13-17 years, the minimum sample size required for each age group was 340, with a precision of ± 5% at 95% confidence level. A total of 1,200 serum samples were collected, comprising 400 in each of the three age groups. The study protocol was approved by the Institutional Review Board of the National University of Singapore.

### EV71-specific neutralizing antibody assay

The serum samples were stored at -20°C, membrane-filtered, and inactivated at 56°C for 30 minutes before use. A modified microneutralization test [23] was used for detecting neutralizing antibody against 5865/Sin/000009, a previously characterized EV71 subgenogroup B4 strain isolated from a fatal case of encephalitis during the local HFMD epidemic in 2000 [24]. Serum sample dilutions of 1:8 to 1:1,024 were assayed, and each dilution was tested in duplicate. Twenty-five microliters of 100 tissue culture infective dose (TCID_50_) of virus was mixed with 25 μl of the appropriate serum dilution, and incubated at 37°C for 2 hours in the presence of CO_2_, followed by the addition of 100 μl of RD rhabdomyosarcoma cell suspension (at 2 × 10^4 ^cells per 0.1 ml). Infected cells and controls were observed with an inverted microscope after 3 days of incubation at 37°C in 5% CO_2_. The highest dilution that prevents the development of cytopathic effect in 50% of the wells was considered as the antibody titer of the sample, and a titer of ≥ 8 was considered positive. Each batch of tests included a positive serum sample of known antibody titer that was relatively reproducible (based on the acceptance criteria of a difference of less than one titer level, with the same titer obtained on most occasions). An antibody-negative serum sample, and uninfected cells also served as controls. The virus titer was determined by virus back-titration.

### Statistical analyses

For EV71 seroprevalence, we calculated the 95% confidence intervals (CI) for binomial proportions using the method of Wilson [25], and their unpaired difference using method 10 described by Newcombe [26]. The geometric mean titer (GMT) of positive sera and corresponding 95% CI were computed by first taking the logarithmic transformation of the titer readings, followed by antilog transformation of the mean and its 95% CI. Comparison of the GMT by age group was performed by first computing the mean and 95% CI of the difference in logarithm-transformed antibody titer, followed by checking if the ratio of 1 was within the confidence limits which had been antilog-transformed [27]. Statistical analyses were carried out using SPSS version 17.0 software (SPSS Inc, Chicago, IL). *P *values of < 0.05 were considered statistically significant.

## Results

The EV71 seroprevalence was 26.9% (95% CI: 24.5--29.5%) among children and adolescents aged 1-17 years in Singapore. It increased significantly from 14.3% (95% CI: 11.2--18.0%) in children aged 1-6 years to 27.8% (23.6--32.3%) in 7-12 year olds, and reached 38.8% (34.1--43.6%) in 13-17 year olds (Table 1). This indicated that the seroconversion rate differed by about 12% between consecutive age groups. The 1996-1997 study revealed that EV71 infection was largely acquired at 2-5 years of age with the seropositive rate increasing at an average of 12% per year in this age range.

**Table 1:** Age-specific prevalence (%) of EV71-specific neutralizing antibody (with 95% confidence intervals) by gender and ethnic group

Demographics	Age group (years)			
	
	1 - 6	7 - 12	13 - 17	1 - 17
**All**	14.3 (11.2--18.0)	27.8 (23.6--32.3)	38.8 (34.1--43.6)	26.9 (24.5--29.5)
				
**Gender**				
Male	13.8 (9.8--19.1)	27.0 (21.6--33.1)	38.0 (29.9--46.9)	24.4 (21.0--28.2)
Female	14.7 (10.4--20.5)	28.7 (22.5--35.9)	39.1 (33.5--44.9)	29.1 (25.7--32.7)
**Ethnic group**				
Chinese	14.7 (11.0--19.3)	22.6 (17.9--28.1)	36.3 (30.7--42.3)	24.3 (21.5--27.5)
Malay	16.1 (9.8--25.2)	40.6 (31.7--50.1)	45.8 (36.2--55.8)	34.9 (29.7--40.6)
Indian	7.5 (2.6--19.9)	27.0 (15.4--43.0)	37.8 (25.1--52.4)	24.6 (17.8--32.9)

No significant gender-specific difference in seroprevalence was observed; 24.4% (95% CI: 21.0--28.2%) of males aged 1-17 years tested EV71-seropositive, compared to 29.1% (95% CI: 25.7--32.7%) among females. The EV71 seroprevalence of Malays at 34.9% (95% CI: 29.7--40.6%) was significantly higher than that of Indians at 24.6% (95% CI: 17.8--32.9%) and Chinese at 24.3% (95% CI: 21.5--27.5%). There was no statistical difference in the seroprevalence between the latter two ethnic groups.

**Figure 1:**
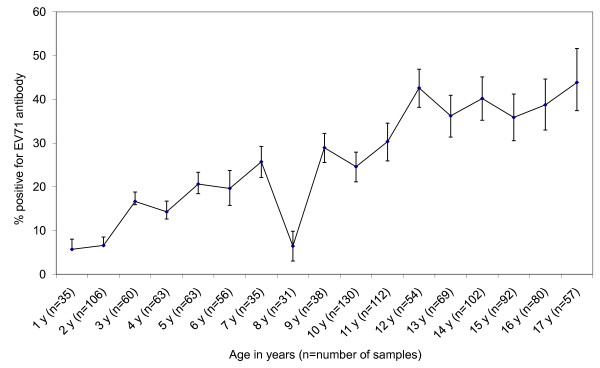
**Prevalence (%) of EV71-specific neutralizing antibody by age. The bars indicate 95% confidence intervals**.

The EV71 antibody prevalence by age showed a general increasing trend except for a dip at the age of 8 years (Figure 1). Children aged 9 years or older had a seroprevalence of at least 25%, and those aged 12 years or older reached a steady state at approximately 40%. In the 1996-1997 study, the EV71 seroprevalence attained a steady state at approximately 50% in the 6-12 year age group [22].

**Figure 2:**
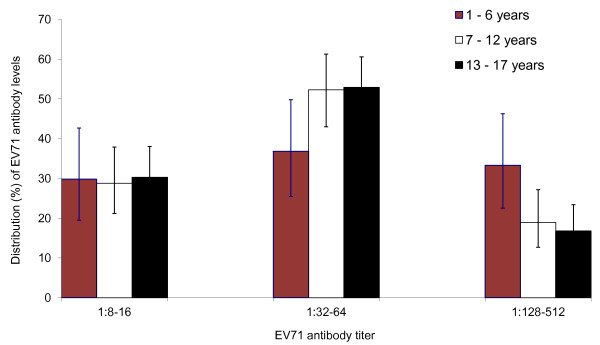
**Distribution (%) of neutralizing antibody titer of EV71-seropositive individuals by age group. The bars indicate 95% confidence intervals**.

Three EV71 antibody titer ranges were defined, i.e. low titer (8-16), moderate titer (32-64) and high titer (128-512). Moderate EV71 antibody levels dominated in all three age groups (Figure 2). About half (49.8%, 95% CI: 44.4--55.3%) of all seropositive subjects displayed moderate levels of EV71 antibody. The proportion of subjects with moderate EV71 antibody levels increased with age, and the difference in proportions (16.1%, 95% CI: 0.9--29.8%) between the age groups of 1-6 years and 13-17 years was significant. On the other hand, the proportion of subjects with high EV71 antibody levels decreased with age, and the differences in proportions between the age group of 1-6 years and the other two older age groups were significant. About one-third (33.3%) of those seropositive in the age group of 1-6 years possessed high EV71 antibody levels, compared to 18.9% in the age group of 7-12 years, and 16.8% in the adolescent group of 13-17 years.

**Figure 3:**
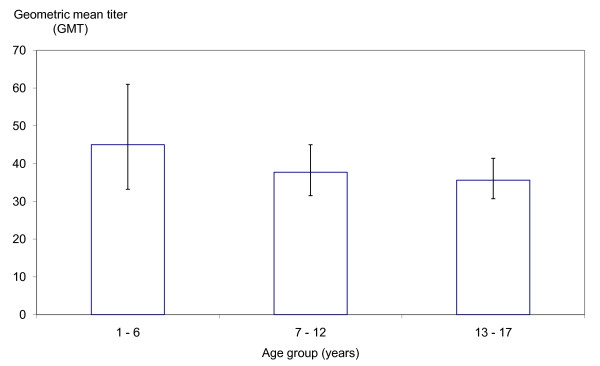
**Geometric mean titer (GMT) of EV71-specific neutralizing antibody by age group. The bars indicate 95% confidence intervals**.

A declining trend in the GMT of seropositive samples was observed across the three age groups (Figure 3). The GMT of EV71 antibody was higher among pre-school children aged 1-6 years (GMT 45.0; 95% CI: 33.2--61.0) than that in those of primary school age (7-12 years old) (GMT 37.7; 95% CI: 31.5--45.0). However, the difference in GMT between these two age groups was not statistically significant (ratio of their GMT was 1.19; 95% CI: 0.84--1.70). The GMT of EV71 antibody among pre-school children aged 1-6 years was also not statistically significantly higher than that among adolescents aged 13-17 years (GMT 35.6; 95% CI: 30.7--41.1), since the ratio of their GMT was 1.26 (95% CI: 0.90--1.77).

In comparison, the 1996-1997 study revealed that the GMT of EV71 antibody was higher among pre-school children aged 2-5 years (GMT 46.8; 95% CI: 34.7--63.1) than those of formal school age (6-12 years) (GMT 28.8; 95% CI: 25.7--32.4) (*P *= 0.002) [22]. The GMT of EV71 antibody appeared to be higher among primary school children aged 7-12 years in our study than those aged 6-12 years in the 1996-1997 study.

## Discussion

Our study revealed that EV71 infection is very common among Singapore children and adolescents, with 39% infected by the time they are in secondary school (i.e. 13-17 years of age). The previous 1996-1997 study was conducted before the first EV71-associated HFMD epidemic occurred in 2000. Our survey commenced in August 2008, several months after an HFMD epidemic started in Singapore around March 2008. This may have contributed in part to the EV71 seroprevalence found in our survey.

Around the same period in 2008, there were heightened levels of EV71 activity in the Asia-Pacific region, including Malaysia, mainland China, Hong Kong, and Taiwan [28]. In China, EV71-associated epidemics were documented in many provinces from March to August 2008, with about 490,000 HFMD cases, including 126 fatalities reported in 2008 [15].

In our study, we only focused on EV71 seroprevalence since serious complications and fatalities in recent HFMD epidemics in the Western Pacific region tend to be generally associated with EV71 than other enteroviruses such as CA16 [29]. In Singapore, 1.8% and 1.6% of the HFMD cases were hospitalized respectively during the EV71-associated HFMD epidemics between March and April 2006, and between April and May 2008. This was more than double the hospitalization rates of the epidemics caused by CA16 (i.e. 0.8% between March and April 2005, and 0.7% between April and May 2007) [21]. EV71 is also more prevalent among severely ill and hospitalized HFMD patients in Taiwan [30,31]. Hospitalization rates increased in Japan in 2000 when EV71 was the main circulating strain [14]. It has been suggested that the clinical outcomes may also be influenced by changes in EV71 neurovirulence and host genetic susceptibility [24,32].

Between 2000 and 2008, there were eight HFMD-related deaths among more than 140,000 HFMD cases notified in Singapore, with a case-fatality rate of lower than 1 per 15,000. The ratio of the number of severe HFMD-related cases (including fatal cases) to the number of HFMD notifications was lower than 1 per 10,000. This suggests that the vast majority of infections are mild or asymptomatic, and complications are rare. The four laboratory-confirmed deaths in 2000 and 2001, and another three serious hospitalized cases including a fatality in 2008 were all associated with EV71.

In Singapore, the HFMD epidemic in 2002 was associated with CA16. Thereafter, there were annual epidemics alternating between CA16 and EV71 from 2005 to 2008 [21]. In Sarawak, Malaysia, a three-year cyclical pattern of EV71-associated epidemics with co-circulation of CA16 was observed to occur in 1997, 2000, 2003, and 2006 [9]. The re-emergence of EV71 as the predominant strain on a three-yearly pattern after 1994 was also noted in Japan [14]. In the past decade, the highest incidence of HFMD in Singapore was 613.4 per 100,000 population in 2008, which was partially associated with EV71. It remains to be seen if EV71-associated HFMD epidemics will occur at two to three year cycles in Singapore, and regular EV71 seroprevalence surveys on children may help to shed light on future trends.

The reasons why EV71 causes frequent and widespread outbreaks in the Asia-Pacific region in the past decade remain unclear [33]. Our findings provide the latest EV71 seroprevalence data in Singapore more than one decade after the previous study in 1996-1997. EV71 seroprevalence among children was observed to be lower in our study compared to that conducted more than 10 years ago, indicating that the pediatric population remains susceptible to EV71 infection. This reduction may be partly attributed to the consistent emphasis on personal hygiene and cleaner environments in pre-schools and schools.

The accumulation of children susceptible to EV71 infection may have triggered the epidemics in Singapore in 2006 and 2008. In addition, the number and capacity of childcare centers have increased rapidly in Singapore over the past decade. The supply and enrollment of childcare centers are expected to continue rising, in view of the emphasis on center-based care, in the form of infant care, childcare and student care centers as an important continuum of care and development for children. With more children congregating in limited spaces, thus providing readily available reservoirs for rapid circulation of the virus, there is a higher risk of transmission to the rest of the population and their family members.

We could make valid comparisons of the findings between our study and the one in 1996-1997, since the same laboratory method was used and serum samples were taken from children attending pediatric hospitals for both studies. Moreover, children with symptoms of HFMD were excluded from both studies.

In our survey, seropositive children aged 12 years (mostly in the last year of primary school education) or older reached a steady proportion of approximately 40%. A declining trend in the GMT of seropositive samples was observed across the three age groups. These observations indicated that transmission was common in pre-schools and primary schools. The GMT of EV71 antibody appeared higher among primary school children aged 7-12 years in our study than that of the 6-12 year age group in the 1996-1997 study. This further suggested that in addition to pre-schools, EV71 transmission has become more frequent in primary schools compared to the past decade. There had also been a corresponding shift in the age distribution of HFMD cases to older children aged 7-12 years old, and the proportion of cases in this age group increased significantly from 7.8% in 2001 to 17.2% in 2008 (*P *< 0.01, χ^2 ^test for trend). On the other hand, while the notifications per 100,000 population remained highest in the pre-school population, the proportion of cases in the age group of 1-6 years decreased significantly from 78.7% in 2001 to 67.6% in 2008 (*P *< 0.01, χ^2 ^test for trend).

The advantages and disadvantages of population-based sampling versus laboratory-based sampling were carefully considered before the commencement of our survey. Laboratory-based sampling was selected as population-based sampling may suffer from unacceptably low response rates due to parental concerns about taking blood samples from their children. The main source of bias in laboratory-based sampling arises when the probability of hospital admission is dependent on the patient's immunity to the disease being studied. To minimize this potential selection bias in our survey, sera of patients who had been diagnosed with HFMD were excluded from the survey.

Singapore has a well-developed healthcare system, and the residual sera were collected from the two main pediatric hospitals in the public sector. Of the hospital discharges among children aged 1-17 years in 2009, 87% were in the public sector. Hence, the selection bias in our study was minimized. Laboratory-based sampling also has an important advantage of avoiding additional discomfort in obtaining blood samples from subjects, particularly from young children [34]. National serosurveys using residual sera from diagnostic laboratories have been carried out to provide seroprevalence estimates of EV71 infection in other countries such as Germany and Vietnam [35,36]. Nonetheless, greater efforts are needed to increase response rates of population-based sampling, e.g. through public education.

## Conclusions

Based on our findings, higher EV71-specific neutralizing antibody titers were observed in pre-school children aged 1-6 years than those in the other two older age groups, indicating that most of the infections had been acquired during early childhood. Seroprevalence surveys conducted periodically to measure the prevalence of EV71-neutralizing antibody in the pediatric population in Singapore will facilitate a more in-depth understanding of the epidemiologic trends and HFMD epidemics associated with EV71 infections.

## Competing interests

The authors declare that they have no competing interests.

## Authors' contributions

LWA, JC, LJ and VTC drafted the manuscript. LWA performed the statistical analyses. MCP and YW carried out the laboratory tests under the supervision of VTC, and contributed to the manuscript. All authors read and approved the final manuscript.

## Pre-publication history

The pre-publication history for this paper can be accessed here:

http://www.biomedcentral.com/1471-2334/11/270/prepub
